# Xylem cavitation susceptibility and refilling mechanisms in olive trees infected by *Xylella fastidiosa*

**DOI:** 10.1038/s41598-019-46092-0

**Published:** 2019-07-03

**Authors:** Erika Sabella, Alessio Aprile, Alessandra Genga, Tiziana Siciliano, Eliana Nutricati, Francesca Nicolì, Marzia Vergine, Carmine Negro, Luigi De Bellis, Andrea Luvisi

**Affiliations:** 1 Department of Biological and Environmental Sciences and Technologies, University of Salento, via Prov.le Monteroni 165, 73100 Lecce, Italy; 2 Department of Physic and Math, University of Salento, via Prov.le Monteroni 165, 73100 Lecce, Italy

**Keywords:** Plant physiology, Environmental sciences

## Abstract

In olive trees, *Xylella fastidiosa* colonizes xylem vessels and compromises water transport causing the olive quick decline syndrome (OQDS). The loss of hydraulic conductivity could be attributed to vessel occlusions induced both by the bacteria biofilm and by plant responses (tyloses, gums, etc.) that could trigger embolism. The ability of the infected plants to detect embolism and to respond, by activating mechanisms to restore the hydraulic conductivity, can influence the severity of the disease symptomatology. In order to investigate these mechanisms in the *X*. *fastidiosa*-resistant olive cultivar Leccino and in the susceptible Cellina di Nardò, sections of healthy olive stems were analysed by laser scanning microscope to calculate the cavitation vulnerability index. Findings indicated that the cultivar Leccino seems to be constitutively less susceptible to cavitation than the susceptible one. Among the vascular refilling mechanisms, starch hydrolysis is a well-known strategy to refill xylem vessels that suffered cavitation and it is characterized by a dense accumulation of starch grains in the xylem parenchima; SEM-EDX analysis of stem cross-sections of infected plants revealed an aggregation of starch grains in the Leccino xylem vessels. These observations could indicate that this cultivar, as well as being anatomically less susceptible to cavitation, it also could be able to activate more efficient refilling mechanisms, restoring vessel’s hydraulic conductivity. In order to verify this hypothesis, we analysed the expression levels of some genes belonging to families involved in embolism sensing and refilling mechanisms: aquaporins, sucrose transporters, carbohydrate metabolism and enzymes related to starch breakdown, alpha and beta-amylase. The obtained genes expression patterns suggested that the infected plants of the cultivar Leccino strongly modulates the genes involved in embolism sensing and refilling.

## Introduction

*Xylella fastidiosa*, a gram-negative bacterium that colonizes xylem vessels, is transmitted to new host plants during sap feeding by insect vectors such as sharpshooter leafhoppers (Hemiptera, Cicadellidae) or spittlebugs (Hemiptera, Cercopidae) and spreads from the site of infection to the plant’s network of xylem vessels^1^. *X*. *fastidiosa* was recognized to be the causal agent of a number of so called leaf scorch diseases such as: Pierce’s disease (PD) of grapevine (*Vitis vinifera*), phony peach disease (PPD) in peach (*Prunus persica*), citrus variegated chlorosis (CVC) or citrus X disease, almond leaf scorch (ALS) in *Prunus amygdalus* and plum leaf scald (PLS) in *Prunus domestica*, coffee leaf scorch (CLS) in *Coffea arabica* and the oleander leaf scorch (OLS) in *Nerium oleander*^2^. Typical severe symptoms affect leaves and fruits causing significant economic loss^2^. Bacterial cells attach to the vessel wall and, by multiplying, they aggregate in a biofilm matrix that includes nucleic acids, proteins, humic substances, and exopolysaccharide (EPS); these biofilms protect bacterial communities from antibiotics, dehydration, host defences, and other stresses and contribute to virulence by allowing the coordinated expression of pathogenicity genes via quorum sensing^3^. It is conventionally thought that this biofilm is the main factor responsible for the blockage of water movement in *X*. *fastidiosa* infected plants; nevertheless, studies have found that vessel occlusions are also caused by the active host plants responses to the presence of the bacterium as defence response^1,3–5^. It was observed that in Pierce’s disease of grapevine a high proportion of colonized vessels in infected leaves were not blocked and instead had small colonies or solitary cells, suggesting that vessel blockage is not a colonization strategy employed by the pathogen but, rather, a by-product of endophytic colonization^1^. Stevenson *et al*.^5^, by following the progression of anatomical symptoms of Pierce’s disease in susceptible *Vitis vinifera* cv. Chardonnay grapevines, reported that leaf and petiole xylem was predominantly occluded with gums and bacteria, whereas, stem xylem was occluded almost exclusively with tyloses. Therefore, gums, pectin gels and *X*. *fastidiosa* biofilms contribute to the vascular obstruction^3^. This loss of xylem water-transporting function determines a decrease in stem-specific hydraulic conductivity. As for other vascular pathogens^6–8^, the phenomenon of vessel cavitation during the disease progression in infected plants could also explain the loss of hydraulic conductivity due to air filling of the vessels.

Pérez-Donoso *et al*.^4^ have reported early embolism in *X*. *fastidiosa*-infected grape and they have correlated this phenomenon with decreased xylem conductivity and drought stress. Nardini *et al*.^9^ summarized the mechanism utilized to restore hydraulic conductivity as the osmotic hypothesis. This hypothesis, proposes an active mechanism that may operate by refilling the embolized vessels with water and the driving force for refilling is generated by the enrichment of the sap with solutes that engages from inorganic and organic solutes (proteins, polysaccharides, etc.), to sugars derived from the hydrolysis of starch (degradation of starch in the parenchyma cells of the xylem produces soluble sugars that are released into the vessels, thereby promoting an osmotic flux of water into their lumen). Recently, *X*. *fastidiosa* was reported under field conditions in Italy (Apulia region), associated with severe cases of Olive Quick Decline Syndrome (OQDS)^10,11^; although the primary role of the bacterium in occlusions formation has been observed^12^, the pathogenesis and symptom formation in olive trees represent research challenges^13^ and mechanisms as vessel embolism induced by the pathogen have never been investigated in this host-pathogen association. The main objective of this work was to evaluate constitutive cavitation susceptibility and activation of refilling mechanisms to restore hydraulic conductivity in olive plants subjected to *Xylella fastidiosa* infection; the comparison between Cellina di Nardò and Leccino, respectively susceptible and resistant olive cultivars^13,14^, aimed to know if cavitation could play a role in determining the severity of symptoms caused by *X*. *fastidiosa*.

## Materials and Methods

### Plant materials and experimental design

Trials were carried out in two orchards near the city of Lecce (Parco Naturale Regionale Bosco e Paludi di Rauccio, 40°27′24.0″N 18°09′43.8″E, Apulia, Italy), in which olive trees of cv. Leccino and Cellina di Nardò were monitored since 2015. Selected plants (25–35 years) were characterized from the same agronomic practices in the last 5 years. Phytosanitary treatments were carried out according to EU Decision 2015/789 which is required to control the insect vector (*Philaenus spumarius*). Orchards were also weekly monitored to detect eventual insect or other pest outbreaks. Tree water status was assessed by Ψw measured every two weeks using a pressure chamber^15^. No irrigation was needed.

Leaf and branches samples were collected from *X*. *fastidiosa*-positive trees (*Xf*-p, 10 naturally infected plants per cultivar; according to visual inspections, the *X*. *fastidiosa*-positive trees of the cv Cellina di Nardò showed the typical symptoms of leaf scorching uniformly distributed in the canopy, instead cultivar Leccino exhibits mild symptoms) and *X*. *fastidiosa*-negative trees, (*Xf*-n, 10 plants/cultivar). The presence of *X*. *fastidiosa* was assessed by polymerase chain reaction (PCR)^16^. In both *Xf*-p and *Xf*-n samples, plants were monitored and tested for some of the most common pathogens in addition to *X*. *fastidiosa*, checking for symptoms caused by natural infection of *Colletotrichum gloeosporioides*, *Mycocentrospora cladosporioides*, *Spilocaea oleagina* and *Pseudomonas savastanoi* pv. *savastanoi*. Diagnostic tests (real-time PCR) were carried out on leaves or woody chips, according to protocols reported in the literature, for *Verticillium dahliae*^17^; *Colletotrichum* spp., *C*. *acutatum*, and *C*. *gloeosporioides*^18^; *Phaeomoniella chlamydospora*^19^; *Phaeoacremonium aleophilum* and *P*. *parasiticum*^20,21^; *Botryosphaeria dothidea*^22^; *Diplodia seriata*^23^ and *Phytophthora* spp.^24^ previously detected in South Italy^25^. *Xf*-p and *Xf*-n trees were negative to every test, apart some sporadic evidence of *C*. *gloeosporioides*, *S*. *oleagina* and *P*. *savastanoi* pv. *savastanoi*. However, we carried out the assessment of xylem vascular diameter and SEM/EDX analysis (see following sub-chapters) just on stem tissues in which these non-systemic pathogens were not achieved. Stem tissues from *Xf*-p and *Xf*-n trees were also used to analyse the expression of genes coding for aquaporins, starch and sucrose metabolism, sucrose transporters, enzymes related to starch breakdown, Alpha and Beta amylase.

### Detection of *X*. *fastidiosa* in planta

Detection of the pathogen *X*. *fastidiosa* was carried out in both the leaves and the branches. Approximately 1 g of leaf petioles (a pool sample from 60 leaves collected from six branches) or vascular bundles (a pool sample from six branches) was transferred to an extraction bag (BIOREBA, Reinach, Switzerland) and 4 ml of extraction buffer (0.2 M Tris – HCl pH 9, 0.4 M LiCl and 25 mM EDTA) were added. Samples were homogenized using a semiautomatic homogenizer (Homex 6, BIOREBA) at 50% maximum speed. DNA extraction was performed following the protocol of Edwards *et al*.^26^. Briefly, the DNA solution is first extracted with a phenol-chloroform–isoamyl alcohol mixture to remove protein contaminants and then precipitated with 100% ethanol.

The PCR protocol with the primers X.fas-0838-a-S-21-X.fas-1439-a-A-19 and XYgyr499-RXYgyr907^16^ was used to confirm the infection of the samples with *X*. *fastidiosa*. Amplification was performed in a 25 μL reaction containing 0.2 μM concentrations of each primer, 200 μM concentrations of dNTPs, 1 x Taq buffer, 2.0 U of Taq DNA polymerase (TaKaRa, Kyoto, Japan), and 100 ng of DNA template. The PCR started with a 3 min denaturation step at 94 °C, followed by 30 cycles of denaturation at 94 °C for 1 min, primer annealing at 55 °C for 30 s, extension at 72 °C for 2 min, and final extension for 7 min.

### Assessment of xylem vessel diameter and vulnerability to cavitation in olive stem

Stem of the *Xf*-n olive trees were excised with sterile razor blades, surface sterilized for 1 min in 70% ethanol and then rinsed three times in sterile distilled water. The cuttings (≈1.5 × 1.5 cm) were fixed in 4% paraformaldehyde in phosphate-buffered saline (1x PBS, hereafter PBS) overnight at room temperature, followed by washing in PBS buffer for 10 min at room temperature. After fixation, samples were dehydrated by two successive 1-h incubations in each of 70, 80, 95, and 100% ethanol, then embedded in paraffin and cut into 30 μm-thick sections with a microtome Leica RM 2155 (Leica Microsystems, Mannheim, Germany). Sections were transferred to 1:1 (v/v) PBS:96% ethanol and maintained at −20 °C until staining. To dissolve the paraffin, sections were embedded in toluene for 3 min at 43 °C. After removing the toluene, all sections were stained with safranin solution (1%) to enhance contrast of wood against void space of vessels. Following the staining, sections were cleared and prepared for microscopic analyses (aqueous embedding on glass slides). All images were taken on a confocal laser-scanning microscope (Carl Zeiss LSM 700 laser scanning microscope, Jena, Germany); all images were acquired with the same microscope settings and analysed using ImageJ v1.46r.

The presented vessel diameter values are the means of more than 400 determinations (arithmetic vessel diameter and the vessel distribution were computed according to Scholz *et al*.^27^); xylem vessels were divided in six classes of diameter (0–15 µm; 15–30 µm; 30–45 µm; 45–60 µm; 60–75 µm; 75–90 µm).

The Student t-test was used to test the statistical significance of the differences found in the vessel density for different classes of diameter observed in the two studied cultivars.

Vulnerability to cavitation (vulnerability index, VI) was calculated using the equation proposed by Carlquist^28^, as follows:

$${\rm{VI}}={\rm{VD}}/{\rm{VF}}$$

VF is vessel frequency (number of vessels per mm^2^, N/mm^2^), while VD is the mean vessel diameter (µm).

### SEM/EDX wood analysis

Stem samples from *Xf*-p and *Xf*-n plant branches were examined with a scanning electron microscope (SEM). Sections handmade using razor blades were dehydrated by critical point step, mounted on aluminum stabs and sputter coated with three layers of gold before the SEM observation with a Tescan Vega LMU model coupled with an energy-dispersed spectrometry (EDS) microanalysis Bruker Quantax 800 (with a lower detection limit per element of less than 0.1%) at an accelerating voltage of 20 kV in high vacuum. An X-Ray spectrum has been collected for 120 sec for every SEM image. This allows us to recognize both the main elements and the trace elements to determine whether there were different chemical substances in the observed structures (tylose, starch, etc); the Student t-test was used to test the statistical significance of the differences found in the atomic percentages of the detected elements. Diameter of the starch granules were measured on SEM micrographs (1000 and 3000×) using software ImageJ 1.46r.

### Total RNA isolation, cDNA synthesis and RT-PCR analysis of gene expression

Stem tissues from *Xf*-p and *Xf*-n plants were frozen in liquid nitrogen and total RNA was isolated from 0.1 gr of samples using TRIZOL (Invitrogen, Carlsbad, USA). cDNA synthesis was carried out using TaqMan® Reverse Transcription Reagents (Applied Biosystems, Foster City, USA) according to the manufacturer’s standard protocol. Amplification reactions were performed using a Biorad CFX96 Real-time PCR cycler and Biorad CFX manager software v3.1 (Bio-Rad, Irvine, CA, USA) using default settings for amplification curve analysis. Each reaction consisted of 13 ng of cDNA, 12.5 μL of Power SYBR Green RT-PCR Master mix (Applied Biosystems), 5.0 µM forward and reverse primers, ultrapure DNase/RNase-free water (Carlo Erba Reagents S.r.l.) in a total volume of 25 μL. The cycling conditions were: 2 min at 50 °C and 10 min at 95 °C, followed by 45 cycles of 95 °C for 15 s and 60 °C for 1 min. Melting curve analysis was performed after PCR to evaluate the presence of non-specific PCR products and primer dimers. Recent models on the embolism sensing and refilling mechanism describe as involved genes the following families: Aquaporins, Sucrose transporters, carbohydrate metabolism and enzymes related to starch breakdown, Alpha and Beta Amylase^29–31^. The expression levels of some of these genes were analysed in stems of the olive trees Leccino and Cellina di Nardò, comparing *Xf*-p and *Xf*-n samples. The used primers were retrieved from the literature or designed with the software Primer Express Software 3.0 on the mRNA sequences deposited in GenBank (Table 1). β*-Actin* and the *Elongation Factor 1-α* that has been previously shown to have stable expression in Leccino stem^32^ and in *O*. *europaea* tissues^33^ were tested and used to normalize the expression levels of the target genes. The Student t-test was used to test the statistical significance of the differences found in the expression profile of genes.

**Table 1:** Primers used for RT-PCR to measure expression levels of analysed genes.

Name	sequence 5′-3′	Reference	GenBank
*Oeβ-Act_F*	ACTATGAACAGGATCTTGAG	Rossi *et al*.^32^	AF545569.1
*Oeβ-Act_R*	GAACCACCACTGAGGACGAT		
*OeEF1-α_F*	CTGACTGCGCCGTCCTTATC	Alagna *et al*.^63^	XM_002527974.1
*OeEF1-α_R*	TGACACCAAGGGTGAAGGC		
*OePIP1_F*	GGCATATAAATCCGGCAGTGA		DQ202708.1
*OePIP1_R*	CGGGTCAACGACAATTTCCT		
*OePIP2_F*	TGCCACCATCCCCATCAC		DQ202709.2
*OePIP2_R*	GATGACAGCAGCTCCAAAGCT		
*OeTIP_F*	CGGCGGCCACGTAAAC		DQ202710.1
*OeTIP_R*	CAATGTGATGTGACCACCAACA		
*Oeβ-Amy_F*	ATATGATGACTACGCCCACGAA		JQ711506.1
*Oeβ-Amy_R*	CGATGCCATCAACATTCAAATG		
*Oeβ-Amy1_F*	TGCCACGATATGATGACTACGC	Alagna *et al*.^63^	unigene01162
*Oeβ-Amy1_R*	TCAGGTTGGAACAAATCCGGGTTC		
*Oeα-Amy2_F*	CGGAGACGCGGTGGAAT		XM_022987933.1
*Oeα-Amy2_R*	CCGTCATTATGAGCATGGTATATTTTT		
*Oeα-Amy_F*	ATCAGGACAGGCATCGGAAT		XM_023038990.1
*Oeα-Amy_R*	GACACTGCCTCCCGCATT		
*OeMST2_F*	GCCAATGTGGACGAGGAGTT		DQ087177.2
*OeMST2_R*	TGCTCCACCTTCCTCGACTCT		
*OeSUT1_F*	TCGGTTATGCGGCTGGAT		JN656245.1
*OeSUT1_R*	CAGGCTTTTGTTTTGGTAAATGG		
*OeSUSy_F*	GCCTGGACTCTACCGAGTTGTT	Alagna *et al*.^63^	unigene02089
*OeSUSy_R*	CACGCATAGGTGTTCCTTGTTC		
*OeINV-V_F*	CCAGTCAGCGAAGTGGAAGAAT	Alagna *et al*.^63^	unigene01665
*OeINV-V_R*	TGTAACCAGCATCAGCATCAGC		
*OeINV-CW_F*	AGACAAGGCAGAGACATTCGAC	Alagna *et al*.^63^	unigene02494
*OeINV-CW_R*	ATGCATCAGAGCACATGAGAAC		
*OeGBSSI_F*	TGTGCCAAAGTCGACCCTGCCG	Alagna *et al*.^63^	unigene00185
*OeGBSSI_R*	TGGTTCACTGCTGGCAGCCCC		

## Results and Discussion

### Xylem vessel diameter and vulnerability to cavitation in stem of Leccino and Cellina di Nardò

**Figure 1:**
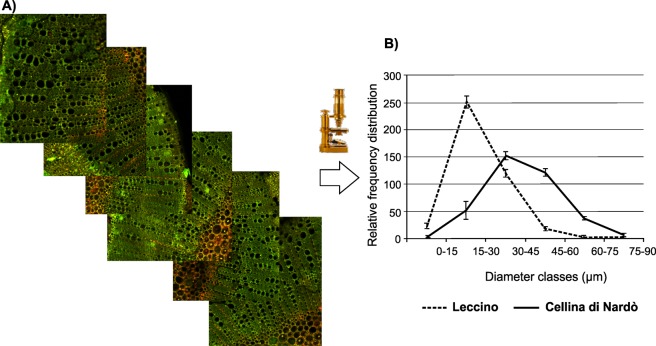
Diameter classes of the xylem vessels in the stems of the cvs. Leccino and Cellina di Nardò. (**A**) Transverse sections of olive stems stained with safranin solution to enhance void space of xylem vessels. (**B**) Distribution of xylem vessels diameter observed in the stem of *Olea europaea* cvs. Leccino and Cellina di Nardò. Please see Table 2 for further statistical analysis.

With regard to the number of vessels per diameter class, Student t-test showed a significant effect of cultivar for the diameter class 0–15 µm (P < 0.05), diameter class 15–30 µm (P < 0.01) and classes 45–60 µm and 60–75 µm (P < 0.001) (Table 2): our results indicated that the cultivar Leccino, reported as the resistant cultivar, displayed a higher number of small diameter vessels (0–15 µm and 15–30 µm) compared to the susceptible cultivar Cellina di Nardò, which displayed a higher number of wide diameter vessels (45–60 µm and 60–75 µm) (Fig. 1). Moreover, the two cultivars differed also for the overall distribution of vessel sizes. The Leccino graph showed a Gaussian distribution curve with a narrow base (median = 26), whereas the base of Cellina di Nardò Gaussian curve is larger (median = 45) (Fig. 1). These data suggested that Leccino cultivar is characterized by a tighter and more homogeneous vessel apparatus if compared to Cellina di Nardò ones.

**Table 2:** T-test outputs of the vessel diameter classes comparison between the stems of the cvs. Leccino and Cellina di Nardò.

	Vessel diameter classes					
	0–15 µm	15–30 µm	30–45 µm	45–60 µm	60–75 µm	75–90 µm
Cultivar	**0**.0319*	**0**.0082**	**0**.**1450**	**0**.0003***	**0**.0005***	**0**.**1261**

*Significance at P < 0.05; **Significance at P < 0.01; ***Significance at P < 0.001.

Previous works speculated on the role of vessel diameter as factor influencing plant’s susceptibility to xylem-dwelling pathogens and several cases have been described where resistance is related to shorter and smaller vessels. Chatelet *et al*.^34^ examined the vascular anatomy of 12 grape varieties as *X*. *fastidiosa*’s hosts and found that tolerant host plants had narrower vessels; they supposed a decrease in the motility of bacteria within the xylem vessels. The implications of xylem structure on the pathogen movement are discussed by Elgersma^35^ that reported length and diameter of xylem vessels as factors in resistance of elms to *Ophiostoma ulmi* (syn. *Ceratocystis ulmi*) due to a limited spreading of the fungus’s spores in the narrowed vessels, so the percentage of vessels with a large diameter was smaller in resistant elms than in susceptible ones. Another focus of the vessels anatomy studies in relation to vascular disease is the cavitation induced by pathogen infections. The loss of hydraulic conductivity could be attributed both to vascular pathogen growth and to plant responses^7^. Tyloses and gels production is a well-known plant defence response to halt the pathogens spreading. Sun *et al*.^36^ showed a quantitative comparison of vascular occlusions in the Pierce’s Disease susceptible versus resistant grapevine genotypes; they found tyloses as the predominant type of occlusion and correlated them with the larger vessels. According to their data, tyloses occlusions does not prevent the pathogen’s systemic spread but they could significantly impede water conduction contributing to disease symptoms development in the susceptible genotypes. Similarly, Pouzoulet *et al*.^37^ described an increased susceptibility to the vascular pathogen *Phaemoniella chlamydospora* in grapevines with wide vessel diameter since fungal compartmentalization was more efficient in wide vessels and also because large vessels displayed a higher quantity of tyloses and occlusions that create a favorable environment for the pathogen’s growth and reduce plant’s hydraulic conductance. Since cavitation assumes a key role in the symptoms development of vascular pathogens as the bacterium *X*. *fastidiosa*, vulnerability to cavitation (vulnerability index, VI) was calculated. Vulnerability index of the Cellina di Nardò showed that this cultivar was almost 2 times more vulnerable than the cultivar Leccino (Table 3).

**Table 3:** Anatomical characters and Vulnerability index (VI) of the olive cultivars Cellina di Nardò and Leccino.

Cultivar	VD (µm)	VF (N/mm^2^)	VI
Cellina di Nardò	**45**.**70 ± 11**.29***	**36**.**60 ± 2**.76***	**1**.**25**
Leccino	**27**.**46 ± 9**.49***	**40**.**70 ± 2**.18***	**0**.**67**

VD = Vessel diameter, VF = Vessel frequency (number of vessels per mm^2^, N/mm^2^), VI = Vulnerability index.

***Significance at P < 0.001.

Table 3 also shows that vessels frequency was lower in the stem of the cultivar Cellina di Nardò.

### SEM/EDX analysis

Against the observed background, the mechanisms capable to refill xylem vessels could be important factors in vascular wilt diseases resistance. Among the embolism repair mechanisms, starch degradation (from parenchyma cells of the xylem to soluble sugars that promotes osmotic water flux capable to refill hydraulic conductivity) is well-known and it is characterized by a dense accumulation of starch grains in the xylem parenchima^38^ that could be revealed by the SEM analysis of the stem cross-sections.

**Figure 2:**
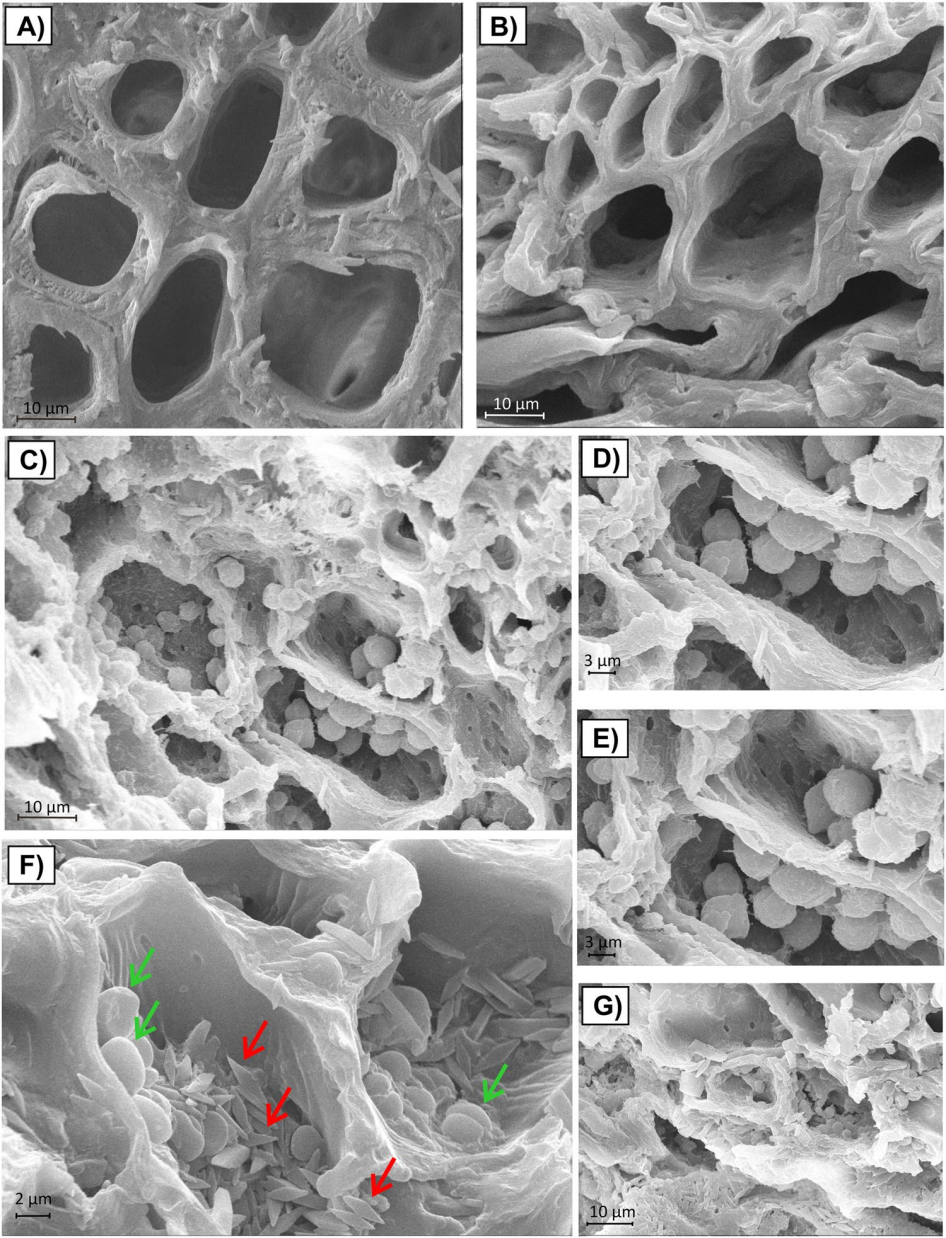
Scanning electron photomicrograph of Leccino and Cellina di Nardò stem cross-sections. (**A**) Stem cross-section of *Xf*-n Cellina di Nardò. (**B**) Stem cross-section of *Xf*-n Leccino. In sections from *Xf*-n samples, vessels appeared free of occlusions. (**C**–**E**) Stem cross-sections of *Xf*-p Leccino; scanning electron photomicrographs of xylem parenchyma reveals a dense accumulation of starch grains. (**F**,**G**) Stem cross-sections of *Xf*-p Cellina di Nardò. In stem’s sections from infected Cellina di Nardò trees, tyloses (indicated by green arrows) and crystals (indicated by red arrows) were observed. *Xf* − n = *X*. *fastidiosa* negative samples; *Xf* − p = *X*. *fastidiosa* positive samples.

In sections from *Xf*-n samples, vessels appeared free of occlusions (Fig. 2A,B).

**Figure 3:**
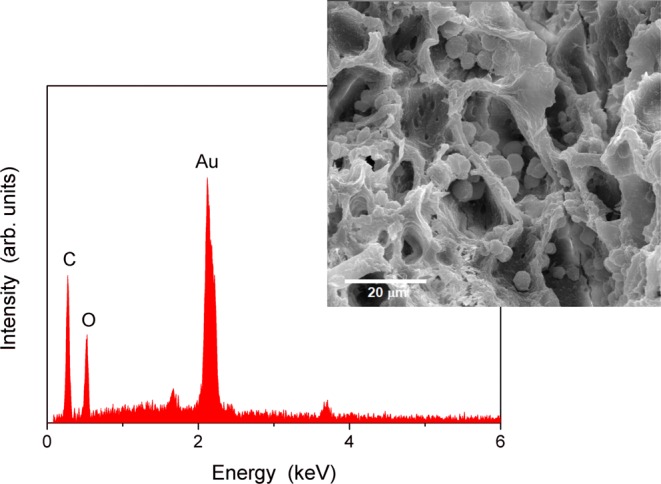
EDX analysis of the starch grains. The SEM/EDX analysis indicated that the structure of the starch grains was composed of carbon and oxygen with atomic percentages that are characteristic of starch according to the data reported in literature^40,41^.

**Figure 4:**
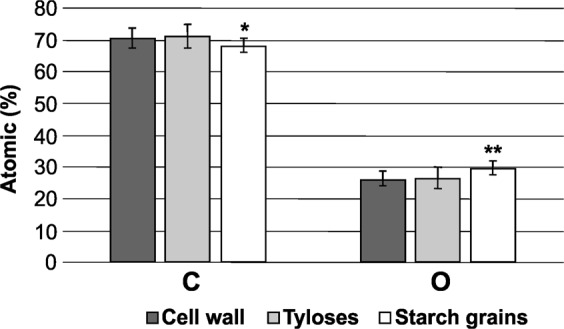
Quantitative results of the elemental analysis of cell wall, tyloses and starch grains. Carbon (C) and oxygen (O) distribution in cell wall, tyloses and starch grains observed in stem cross-sections of Leccino and Cellina di Nardò. The atomic percentages of C and O in the observed grains showed statistically significant differences in comparison with the cell wall composition of the xylem vessel according to Student t-test (*P < 0.05; **P < 0.01). The observed masses hypothesized to be tyloses exhibited no differences when compared with the cell wall composition.

Only in the cross-sections from the stems of infected Leccino were observed starch grains (Fig. 2C–E); they appeared as spherical structures with an average diameter of 4.01 ± 1.02 µm. Figure 3 shows the result of the elemental analyses of the starch grains conducted by SEM/EDX. Since starch is constituted by glucose units, the presence of the three elements carbon (C), hydrogen (H) and oxygen (O) would be expected; however, only C and O can be detected; H is not distinguished as there are no core electrons in hydrogen but only valence electrons. In addition, the signals emitted from H 1 s valence electrons would overlap with signals of other valence electrons and would not be possible for identification^39^. The atomic percentages of C and O in the observed grains gave an elemental signature that is characteristic of starch according to the data reported in literature^40,41^ and it showed statistically significant differences in comparison with the cell wall composition of the xylem vessel (Fig. 4).

In stem’s sections from infected Cellina di Nardò trees, tyloses and crystals were observed (Fig. 2F,G). Tyloses differed from starch grains for size and composition; tyloses were found in some vessels as masses of different sizes (the average diameter, assuming these structures as circles, is 2.04 ± 0.60 µm; tyloses size showed a statistically significant difference in comparison with starch grains according to Student t-test, P < 0.001) and sometimes they appeared as partially included in the cell wall of the vessel’s lumen; about the composition, the energy-dispersive X-ray (EDX) analysis showed no differences between the cell wall composition and the observed masses (Fig. 4); this is reasonable since tyloses are outgrowths on parenchyma cells of xylem vessels^42^.

Several studies have documented a dense accumulation of starch grains in the vessel’s cells as an integral part of embolism recovery: Masrahi *et al*.^43^ observed in xylem parenchima of the two lianas *Cocculus pendulus* and *Leptadenia arborea* a great quantity of starch grains that can reduce the solute potential of xylem sap improving the upward flow of water, this proposed strategy could help plants to repair air filled vessels and to survive in the water deficit of their severe habitat. Starch granules are readily apparent in the fibers of grapevine xylem and it was proposed that they generated an osmotic driving force for refilling^38^. Bucci *et al*.^44^ explained that the abundant starch granules observed in the vessels of the two savanna tree species *Schefflera macrocarpa* and *Caryocar Brasiliense* could intensificate vascular water uptake by increasing osmotically active solutes. Finally, according to Nardini *et al*.^9^, starch depolymerization is apparently activated during xylem refilling and treatments that repress refilling also inhibit starch hydrolysis both in the lab and in field-grown plants.

According to our obtained data, the cultivar Leccino seems to be less susceptible to cavitation and able to activate more efficient refilling mechanisms restoring vessel’s hydraulic conductivity.

### Expression of gene families involved in embolism sensing and refilling mechanism

**Figure 5:**
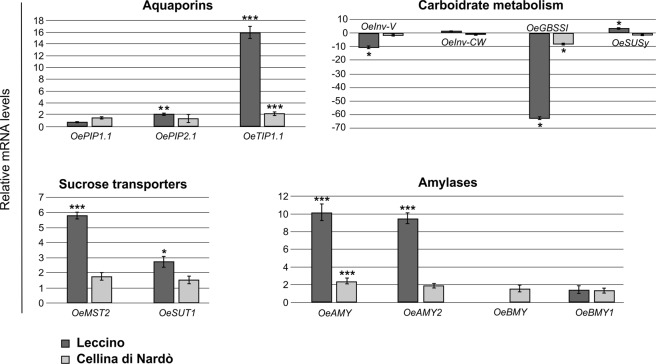
Expression of genes involved in embolism sensing and refilling mechanisms in *X*. *fastidiosa* infected Leccino and Cellina di Nardò plants. Quantitative analyses of expression of genes coding for aquaporins, sucrose transporters, enzymes of the carbohydrate metabolism and enzymes related to starch breakdown, Alpha and Beta Amylase. *OePIP1*.*1* and *OePIP2*.*1* = *Olea europaea Plasma Membrane Intrinsic Protein 1*.*1* and *2*.*1*; *OeTIP1*.*1* = *Olea europaea Tonoplast Intrinsic Protein 1*.*1*; *OeInv-V* = *Olea europaea Vacuolar Invertase*; *OeInv-CW* = *Olea europaea Cell Wall Invertase*; *OeGBSSI* = *Olea europaea Granule-Bound Starch Synthase I*; *OeSusy* = *Olea europaea Sucrose Synthase*; *OeMST2* = *Olea europaea Monosaccharide Transporter 2*; *OeSUT1* = *Olea europaea Sucrose Transporter 1*; *OeAMY* and *OeAMY2* = *Olea europaea α-Amylases*; *OeBMY* and *OeBMY1* = *Olea europaea β-Amylases*. The significant differences were highlighted according to Student t-test (*P < 0.05; **P < 0.01; ***P < 0.001).

In order to verify the suggested hypothesis of a more efficient strategy to restore vessel’s conductivity in the cultivar Leccino, we analysed the expression levels of the gene families involved in embolism sensing and refilling mechanism. In the Fig. 5 are reported the expression levels of the genes coding for three aquaporins: *OePIP1*.*1*, *OePIP2*.*1* and *OeTIP1*.*1*. Aquaporins belong to a highly conserved group membrane major intrinsic protein (MIPs), they are found in the cell membranes of all living organisms and allow water flux across membranes^45^. In plants, the aquaporins can increase the hydraulic conductivity of the membrane by 10- to 20-fold^46^. In the two analysed cultivars, the *OePIP1*.*1* transcript did not significantly change in response to the *X*. *fastidiosa* infection; *OePIP2*.*1* resulted upregulated in the cultivar Leccino (fold change = 2.04 ± 0.17) whereas in the cultivar Cellina di Nardò its level didn’t result significantly modified. According to Secchi *et al*.^47^ that conducted experiments in a heterologous system like *Xenopus* oocyte, it has been observed that *Oe*PIP2.1 water transport activity is much more effective than that of *Oe*PIP1.1. *OeTIP1*.*1* resulted strongly upregulated in the cultivar Leccino (fold change = 15.92 ± 1.02) and upregulated also in the stems of the infected Cellina di Nardò (fold change = 2.16 ± 0.31) indicating a plant disease response. According to the obtained data, in the cultivar Leccino, the genes coding for the aquaporins showed more interesting expression profiles in *X*. *fastidiosa* infected stems and *OeTIP1*.*1* deserve specific attention. Secchi *et al*.^47^ investigated the tissue-specific expression of these three aquaporin genes and found that *OeTIP1*.*1* was the aquaporin with a stem-specific expression while in root and leaves the expression was low. In the Fig. 5 expression data of the genes coding for the following α- and β-amylases and sugar transporters are reported: *OeAMY*, *OeAMY2*, *OeBMY*, *OeBMY1*, *OeSUT1* and *OeMST2*. In the stems of the cultivar Cellina di Nardò, only the gene *OeAMY* was upregulated in the *X*. *fastidiosa* infected trees (fold change = 2.42 ± 0.35); whereas, in the stems of the infected Leccino trees, were upregulated the genes: *OeAMY* (fold change = 10.20 ± 0.92), *OeAMY2* (fold change = 9.51 ± 0.62), *OeSUT1* (fold change = 2.74 ± 0.35) and *OeMST2* (fold change = 5.79 ± 0.22). According to Secchi *et al*.^30^, that studied transcriptome response to embolism formation in stems of *Populus trichocarpa*, the upregulation of amylases and sugar transporters were among the major biological activities associated with the proposed response of parenchyma cells to embolism. The proposed refilling mechanism is linked to starch to sugars conversion that increases the solute concentration of the residual water in the vessel’s lumens thus promoting xylem refilling by altering the osmotic potential^29,30^. The strongly upregulation of the genes coding for α-amylases and sugar transporters in the stems of infected Leccino trees could be correlated to an active response to restore hydraulic transport capacity during vessel cavitation as previously proposed for other plant species^48–50^.

The ability to activate refilling mechanisms in response to cavitation requires efficient mechanisms of embolism sensing. Secchi and Zwieniecki^51^ proposed the sucrose as stimulus to sense embolism; in fact, they demonstrated that the only presence of sucrose activated physiological and molecular response similar to that induced by embolism (upregulation of aquaporins, amylases and sugar transporters family). Therefore, sucrose is a central molecule for sensing during plant defence against pathogens; there are two alternative routes of sucrolytic carbohydrate mobilization: one is reversible and is catalyzed by the sucrose synthase (SUSY); the other route is the irreversible hydrolytic cleavage by invertases (INVs). There are three groups of INV enzymes: the alkaline/neutral invertases (A/NInv) localized in cytosol, mitochondria and/or in plastids and two types of acid invertases, the insoluble one bound to the cell wall (cell wall invertase, CWI) and a soluble one found in the vacuole space (vacuolar invertase, VI); the alkaline/neutral invertases are involved in plant growth and development while the acid invertases are also modulated during plant infection^52^. The expression levels of the genes *OeSusy*, *OeInv-CW*, *OeInv-V* in the stems of infected Leccino and Cellina di Nardò trees are reported in the Fig. 5.

The level of expression of *OeSusy* was higher (fold change = 3.13 ± 0.30) only in stems of infected Leccino plants (Fig. 5). The genes coding for the acid invertases, *OeInv-CW* and *OeInv-V* resulted respectively stable in infected plants in comparison to healthy ones of the two cultivars (Fig. 5) while *OeInv-V* was strongly downregulated in the cultivar Leccino (fold change = −10.47 ± 0.89).

The activity of the sucrose synthase and of the acid invertases were suggested to be involved in plant stress responses and signaling cascades (reviewed by Bolouri-Moghaddam *et al*.^53^). *Susy* transcript levels increased in nematode infected *Arabidopsis thaliana* plants^54^ and in different *Vitis vinifera* varieties undergoing phytoplasma infection^55^.

Further, the expression of the different INVs was found to be related to plant defence: a reduction of *Inv-V* expression has been observed during the infection of *Vicia faba* by *Uromyces fabae* and *Vitis vinifera* by *Erysiphe necator* and *Plasmopara viticola*^56,57^. This down-regulation was attributed to a decrease in the availability of sucrose in the storage compartment^56,57^. Other works reported a decrease in the starch content during pathogen infection suggesting that the degradation of starch provides more substrates to sucrose synthesis^52^. Interestingly, the gene coding for GBSSI (Granule-Bound Starch Synthase I), the most important enzyme in storage starch biosynthesis^58^ resulted strongly downregulated both in the stems of Leccino (fold change = −62.61 ± 1.14) and in the Cellina di Nardò (fold change = −7.98 ± 0.42) infected trees (Fig. 5); this inhibition of the *OeGBSSI* expression could indicate the block of the conversion of sucrose to starch, supporting the sucrose’s role as signaling molecule, or its involvement in other plant defence mechanisms. Sure enough, Wall *et al*.^59^ reported GBSSI as a protein related to defence and in the work of Yi *et al*.^60^ *GBSSI* resulted among the downregulated gene in rice plants infected with *Xanthomonas oryzae* pv. *oryzae*. Thus, also in relation to the mechanisms that involves sucrose as important signal molecule for embolism and plant defence response, in the infected plants of the cultivar Leccino the transcript levels of the above genes showed more interesting expression patterns. Moreover, sucrose has been described as an inducer of the phenylpropanoid pathway^61^ that leads to the biosynthesis of lignin which has already been associated with the major *X*. *fastidiosa* resistance of the cultivar Leccino^62^.

## Conclusion

Here we reported, for the first time, the measurement of the xylem vessels diameter in the two *Olea europaea* cultivars Leccino and Cellina di Nardò; these anatomical features, as described in the previous sections, may provide an indication of susceptibility/resistance to *Xylella fastidiosa*. In the *Olea europaea* plants, as for the other plant species affected by *X*. *fastidiosa*, the symptoms development is correlated with a decrease of xylem water-transporting function that could cause embolism. We hypothesized that differences in cavitation susceptibility determined by the constitutive anatomy of the xylem and a major capacity to active refilling mechanisms after embolism sensing could be involved in the specific cultivar response to the pathogen.
